# Soil respiration and its response to climate change and anthropogenic factors in a karst plateau wetland, southwest China

**DOI:** 10.1038/s41598-024-59495-5

**Published:** 2024-04-15

**Authors:** Hongyu Jia, Xuehai Fei, Jingyu Zhu, Weiduo Chen, Rui Chen, Zhangze Liao, Binghuang Zhou, Yingqian Huang, Haiqiang Du, Peng Xu, Xu Zhang, Wangjun Li

**Affiliations:** 1 College of Resources and Environmental Engineering, Key Laboratory of Karst Georesources and Environment (Guizhou University), Ministry of Education, Guizhou University, 2708 Huaxi Avenue, Guiyang, 550025 Guizhou China; 2 Guizhou Karst Environmental Ecosystems Observation and Research Station, Ministry of Education, Guiyang, 550025 Guizhou China; 3 Guizhou Provincial Double Carbon and Renewable Energy Technology Innovation Research Institute, Guiyang, 550025 Guizhou China; 4 Guizhou Caohai Observation and Research Station for Wet Ecosystem, National Forestry and Grassland Administration, Weining, 553100 Guizhou China; 5 Guizhou Province Key Laboratory of Ecological Protection and Restoration of Typical Plateau Wetlands (Guizhou University of Engineering Science), Bijie, 55170 Guizhou China

**Keywords:** Soil CO_2_ emission, DNDC model, Temperature, Precipitation, Anthropogenic factors, Yunnan-Guizhou Plateau wetland, Ecological modelling, Wetlands ecology, Climate-change ecology

## Abstract

It is important to investigate the responses of greenhouse gases to climate change (temperature, precipitation) and anthropogenic factors in plateau wetland. Based on the DNDC model, we used meteorological, soil, and land cover data to simulate the soil CO_2_ emission pattern and its responses to climate change and anthropogenic factors in Guizhou, China. The results showed that the mean soil CO_2_ emission flux in the Caohai Karst Plateau Wetland was 5.89 ± 0.17 t·C·ha^−1^·yr^−1^ from 2000 to 2019, and the annual variation showed an increasing trend with the rate of 23.02 kg·C·ha^−1^·yr^−1^. The soil total annual mean CO_2_ emissions were 70.62 ± 2.04 Gg·C·yr^−1^ (annual growth rate was 0.28 Gg·C·yr^−1^). Caohai wetland has great spatial heterogeneity. The emissions around Caohai Lake were high (the areas with high, middle, and low values accounted for 3.07%, 70.96%, and 25.97%, respectively), and the emission pattern was characterized by a decrease in radiation from Caohai Lake to the periphery. In addition, the cropland and forest areas exhibited high intensities (7.21 ± 0.15 t·C·ha^−1^·yr^−1^ and 6.73 ± 0.58 t·C·ha^−1^·yr^−1^, respectively) and high total emissions (54.97 ± 1.16 Gg·C·yr^−1^ and 10.24 ± 0.88 Gg·C·yr^−1^, respectively). Croplands and forests were the major land cover types controlling soil CO_2_ emissions in the Caohai wetland, while anthropogenic factors (cultivation) significantly increased soil CO_2_ emissions. Results showed that the soil CO_2_ emissions were positively correlated with temperature and precipitation; and the temperature change had a greater impact on soil respiration than the change in precipitation. Our results indicated that future climate change (increased temperature and precipitation) may promote an increase in soil CO_2_ emissions in karst plateau wetlands, and reasonable control measures (e.g. returning cropland to lakes and reducing anthropogenic factors) are the keys to controlling CO_2_ emissions.

## Introduction

The greenhouse effect caused by greenhouse gases (GHGs) and a series of environmental and climate problems caused by the greenhouse effect has attracted the attention of scientists worldwide^1–5^. CO_2_ is the most important atmospheric GHGs and profoundly impacts global climate change^6–8^. The latest data released by the IPCC show that the average annual emissions of each group of GHGs from 2010 to 2019 were greater than those in any previous decade (45 ± 5.5 Gt CO_2_ emissions in 2019)^9^. Massive GHGs emissions have led to global changes in the land, ocean, and atmosphere. The WMO reported that the current average global temperature (2022) was far above the preindustrial temperature (1.15 ℃)^10^. Currently, the frequent occurrence of extreme weather events also confirms that global warming caused by increasing GHGs emissions will not only worsen our living spaces and conditions but also threaten the safety of ourselves and plants and animals living together on Earth^5,11,12^. Therefore, the issue of GHGs emissions is the focus of environmental problems. Studying GHGs emission patterns and their response mechanisms to various factors is particularly important.

The carbon cycle flux during soil respiration is high and is affected by climate change and anthropogenic factors^4,13,14^. Soil respiration, which is the way for soil carbon to return to the atmosphere, is a major part of the C cycle flux in terrestrial ecosystems. This process includes autotrophic respiration of plant roots, heterotrophic respiration correlated to root carbon, litter from plant roots and leaves, and decomposition of soil organic matter as well^15^. Soils store two-thirds of the carbon in the whole ecosystem^16,17^. Such a large amount of C storage gives the soil the ability to affect atmospheric CO_2_ concentrations and global climate change^15^. Similarly, soil respiration can be affected by climate change, LULC (land use/cover) change, and anthropogenic factors, leading to changes in soil respiration intensity and thereby changes in the amount of CO_2_^18–21^. A study of the northern Tibet alpine grassland revealed that the temperature rising of 3.74 °C caused an 86.86% increase in soil CO_2_ emissions^22^. In semiarid grassland areas, a decrease in precipitation resulted in a 43% reduction in soil CO_2_ emissions, and the increased precipitation resulted in a 75% reduction^23^. Over the past 150–300 years, land use/cover changes have caused losses of 100 ~ 200 Pg C^24^. Nitrogen addition caused soil respiration to increase by 14% in semiarid alfalfa pastures in the Northwest Territories within two years^25^. The soil carbon content increased by 16% in non-grazed and burned areas and decreased by 38% in burned and overgrazed areas^18^. Many studies have confirmed that climatic conditions, land use/cover change, and anthropogenic factors are the causes of soil CO_2_ emission changes. However, if we clarify the mechanism of these influencing factors on soil CO_2_ emissions, we can reduce soil CO_2_ emissions through reasonable methods or means to achieve the purpose of controlling GHGs emissions, preventing further global warming trends, and realizing sustainable development of human society.

The extreme environment and relatively harsh hydrothermal and soil conditions in high-altitude areas have led to current limitations in the study of carbon dynamics^26^. For this reason, high-altitude ecosystems are very sensitive to external changes and are susceptible to climate change and other factors, making them natural laboratories for studying global change and its impacts^27,28^. The ecological processes of plateau wetlands are complex and sensitive to global climate change. The freeze–thaw effect and wetland biological complexity cause the intensity and pattern of soil CO_2_ emissions and its major controlling factors to be uncertain^29,30^. At present, most studies on soil carbon dynamics in plateau wetlands have focused on the Qinghai‒Tibet Plateau^31–33^. As a representative karst wetland ecosystem on the Yunnan-Guizhou Plateau, there are few reports on soil respiration in the Caohai wetland. However, the spatiotemporal pattern of soil CO_2_ emissions in this wetland area and its response to climate change are unclear. Therefore, the Caohai Karst Plateau wetland in Guizhou Province was chosen. Our purpose was to (1) clarify the magnitude, spatiotemporal pattern, and trend of soil CO_2_ emissions in karst plateau wetlands, and (2) clarify the influences of anthropogenic factors and meteorological factors (temperature and precipitation) on wetland soil CO_2_ emissions.

## Results

### Intensity and interannual variation characteristics

**Figure 1:**
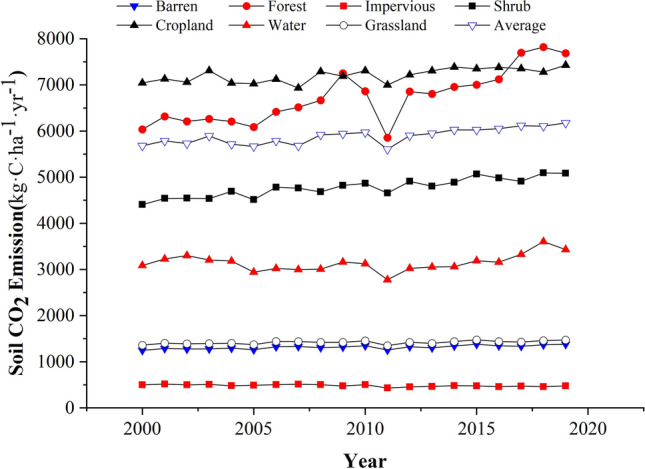
Interannual variations of soil CO_2_ emission fluxes.

Interannual changes in CO_2_ emissions from 2000 to 2019 (Fig. 1) revealed that the average soil CO_2_ emission in the past 20 years was 5.89 ± 0.17 t·C·ha^−1^·yr^−1^ (5.61–6.18 t·C·ha^−1^·yr^−1^, reaching the lowest and highest values in 2011 and 2019, respectively). The average total CO_2_ emission (approximately 12 thousand hectares) was 70.62 ± 2.04 Gg·C·yr^−1^ (74.09 ~ 67.25 Gg·C·yr^−1^). The overall CO_2_ emission flux and total CO_2_ emissions exhibited significant fluctuating trends, with the CO_2_ emission flux increasing at 23.02 kg·C·ha^−1^·yr^−1^ and the total CO_2_ emission increasing at 0.28 Gg·C·yr^−1^. Both showed a relatively obvious downward trend in 2011, which may be due to the significant decrease in temperature in 2011. From the perspective of the CO_2_ emission fluxes of each LC type, the average CO_2_ emissions of cropland were the highest, while those of impervious land were the lowest. The variation in the average CO_2_ emission flux of each LC type was as follows: cultivated cropland (7.21 t·C·ha^−1^·yr^−1^) > forest (6.73 t·C·ha^−1^·yr^−1^) > shrub (4.78) t·C·ha^−1^·yr^−1^) > grassland (3.14 t·C·ha^−1^·yr^−1^) > water (1.42 t·C·ha^−1^·yr^−1^) > barren land (1.31 t·C·ha^−1^·yr^−1^) > impervious land (0.48 t·C·ha^−1^·yr^−1^).

**Figure 2:**
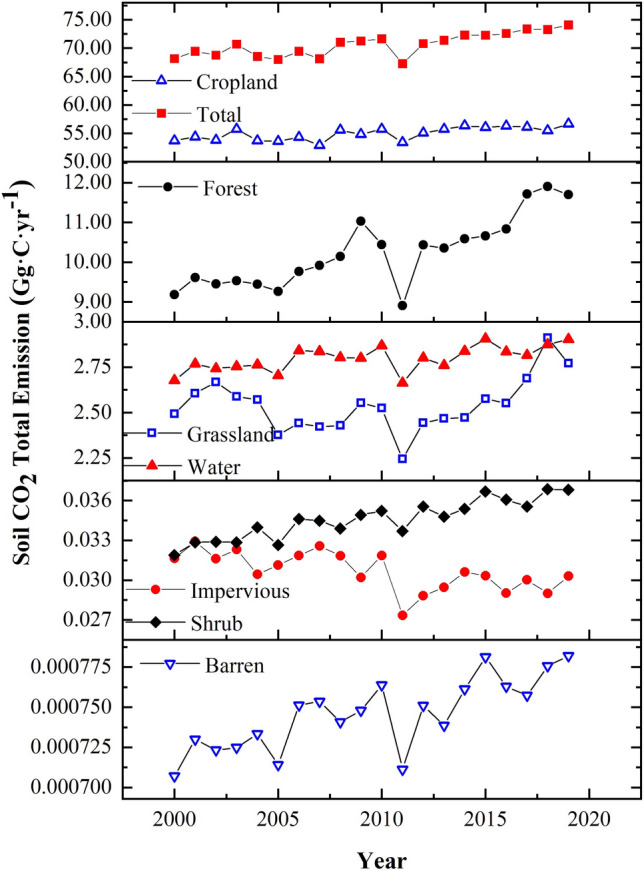
Interannual variation characteristics of the total soil CO_2_ emissions.

Results showed that the average total CO_2_ emission from cropland was the highest (54.97 Gg·C·yr^−1^), accounting for 77.8% of the total (70.62 Gg·C·yr^−1^), and the average total CO_2_ emissions from barren land were the lowest (1.00 t·C·yr^−1^) and were almost negligible (Fig. 2 and Table 1). The variations in average total CO_2_ emissions for each LC type were as follows: cropland (54.97 Gg·C·yr^−1^) > forest (10.24 Gg·C·yr^−1^) > water (2.80 Gg·C·yr^−1^) > grassland (2.54 Gg·C·yr^−1^) > shrub (0.035) Gg·C·yr^−1^) > impervious (0.031 Gg·C·yr^−1^) > barren (0.001 Gg·C·yr^−1^) (Table 1).

**Table 1:** Quarterly variation characteristics of soil CO_2_ emission (unit: t·C·ha^−1^·season^−1^).

Quarter	Spring	Summer	Autumn	Winter
Grassland	1.54	1.43	1.08	0.70
Impervious	0.12	0.19	0.12	0.09
Cropland	3.08	2.42	2.58	1.12
Shrub	0.88	1.88	1.16	0.54
Barren	0.24	0.48	0.36	0.19
Forest	2.68	2.82	1.97	1.38
Water	0.27	0.52	0.39	0.20
Average	1.26	1.39	1.10	0.60
Standard deviation	1.22	1.03	0.91	0.50

During the study period (2000 ~ 2019), except for impervious land, the other LC types exhibited an upward trend. The soil CO_2_ emissions of Caohai wetland increased by 23.02 kg·C·ha^−1^·yr^−1^ (Fig. 2, Supplementary Table S1). In terms of the emission flux, the increase patterns of the different LC types were as follows: forest (81.62 kg·C·ha^−1^·yr^−1^) > shrub (31.55 kg·C·ha^−1^·yr^−1^) > cropland (17.85 kg·C·ha^−1^·yr^−1^) > grassland (10.17 kg·C·ha^−1^·yr^−1^) > barren land (5.28 kg·C·ha^−1^·yr^−1^) > water (3.80 kg·C·ha^−1^·yr^−1^) > impervious water (2.51 kg·C·ha^−1^·yr^−1^). In terms of total emissions, the variation in the increase amplitude was as follows: cropland (136 t·C·yr^−1^) > forest (124 t·C·yr^−1^) > grassland (8 t·C·yr^−1^) > water (7 t·C·yr^−1^) > shrub (0.228) t·C·yr^−1^) > impervious (0.159 t·C·yr^−1^) > barren (0.003 t·C·yr^−1^).

In general, the Caohai wetland soil system is a carbon "source", and the soil CO_2_ emissions are increasing annually. From the results, we can conclude that the average values of both emission flux and total emission from cropland are the highest among all LC types in Caohai. Compared with other LC types, cropland is more affected by human factors, and its CO_2_ emissions are more likely to be affected by human control. Therefore, for the purpose of reducing emissions and controlling global warming, reasonable planning of cropland areas, adopting reasonable field management measures and farming patterns will be positive in controlling soil CO_2_ emissions.

### Annual variation trend

**Figure 3:**
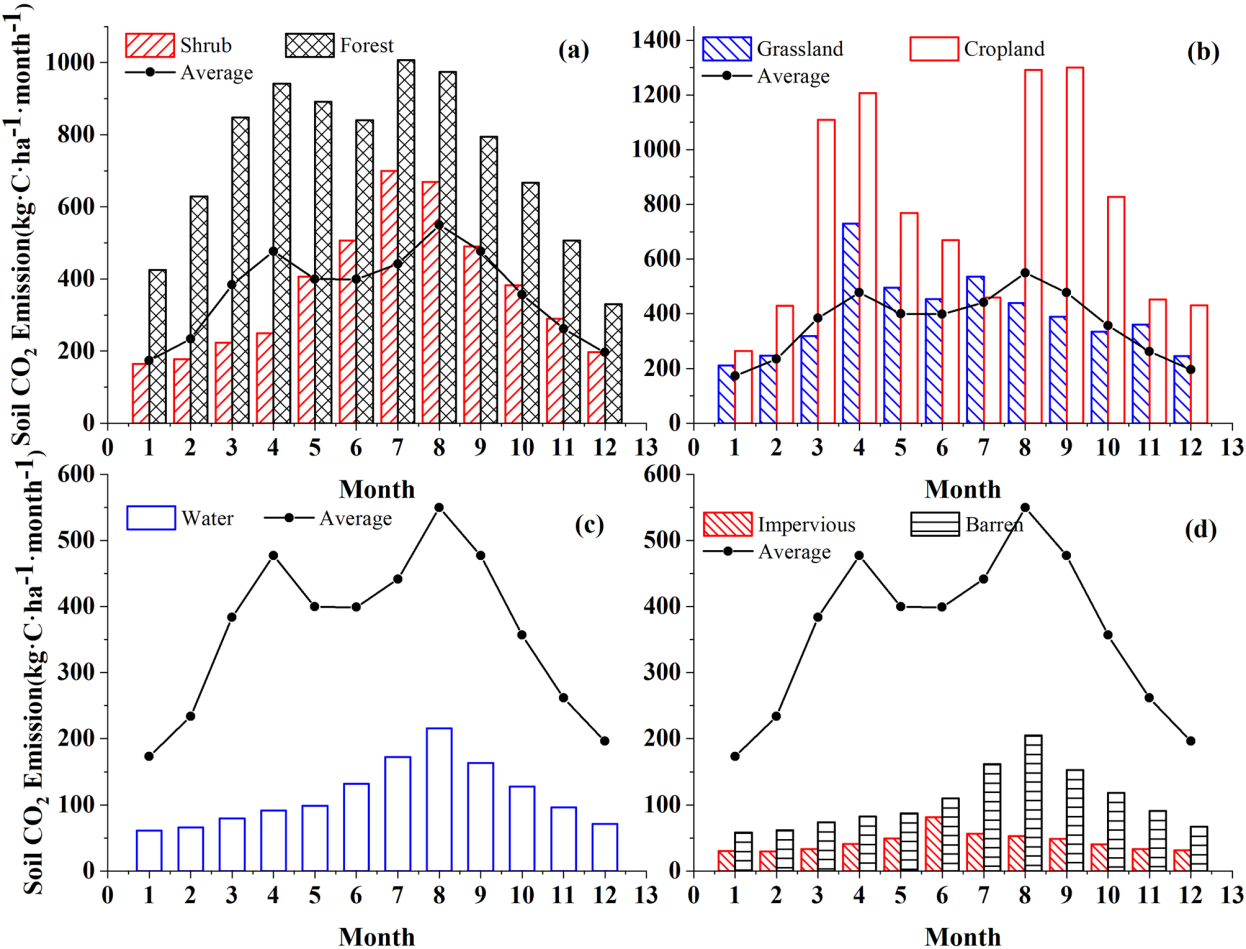
Monthly variation characteristics of soil CO_2_ emission.

The annual variation characteristics of soil CO_2_ emission fluxes in Caohai wetland as a whole and each land cover type are shown in Fig. 3 and Table 1. From the perspective of the Caohai wetland overall at the monthly scale, the average CO_2_ emission flux was 368.08 ± 339.11 kg·C·ha^−1^·month^−1^, and the CO_2_ emission pattern exhibited a bimodal curve. The month with the highest emission flux was August, and the month with the lowest emission flux was January (173.13 kg·C·ha^−1^·month^−1^). From the perspective of each LC type, the emission patterns of cropland, forest, and grassland were consistent with the overall trend of the Caohai wetland, showing a bimodal curve pattern. Among the three LC types, the CO_2_ emission flux reached one peak in April, while the other peak appeared in September in cropland and in July in forest and grassland. The CO_2_ emission fluxes of shrubs, barren areas, forests, water bodies, and impervious areas exhibited unimodal curve patterns. The variation in the maximum emissions of each LC type was as follows: cropland (1299.92 kg·C·ha^−1^·month^−1^) > forest (1007.11 kg C·ha^−1^·month^−1^) > grassland (729.01 kg C·ha^−1^·month^−1^) > shrub (699.24) kg C·ha^−1^·month^−1^) > water (215.98 kg C·ha^−1^·month^−1^) > barren (204.94 kg C·ha^−1^·month^−1^) > impervious (81.18 kg C·ha^−1^·month^−1^). The variations in the minimum emissions of each LC type were as follows: forest (329.62 kg·C·ha^−1^·month^−1^) > cropland (263.52 kg·C·ha^−1^·month^−1^) > grassland (210.47 kg·C·ha^−1^·month^−1^) > shrub (164.06 kg·C·ha^−1^·month^−1^) > water (61.32 kg C·ha^−1^·month^−1^) > barren (57.96 kg C·ha^−1^·month^−1^) > impervious (29.32 kg C·ha^−1^·month^−1^).

From the perspective of the Caohai wetland overall at the seasonal scale, the pattern exhibited a unimodal curve distribution with high in summer and low in winter. The variation in seasonal emissions was as follows: summer (1.39 t·C·ha^−1^·season^−1^) > spring (1.26 t·C·ha^−1^·season^−1^) > autumn (1.10 t·C·ha^−1^·season^−1^) > winter (0.60 t·C·ha^−1^·season^−1^). From the perspective of each LC type at the seasonal scale, the variation of forest, shrub, water, barren land, and impervious land was consistent with that of Caohai as a whole. The variation in grassland and cultivated land showed that emissions were greatest in spring and lowest in winter.

### Spatial variation of CO_2_ emissions

**Figure 4:**
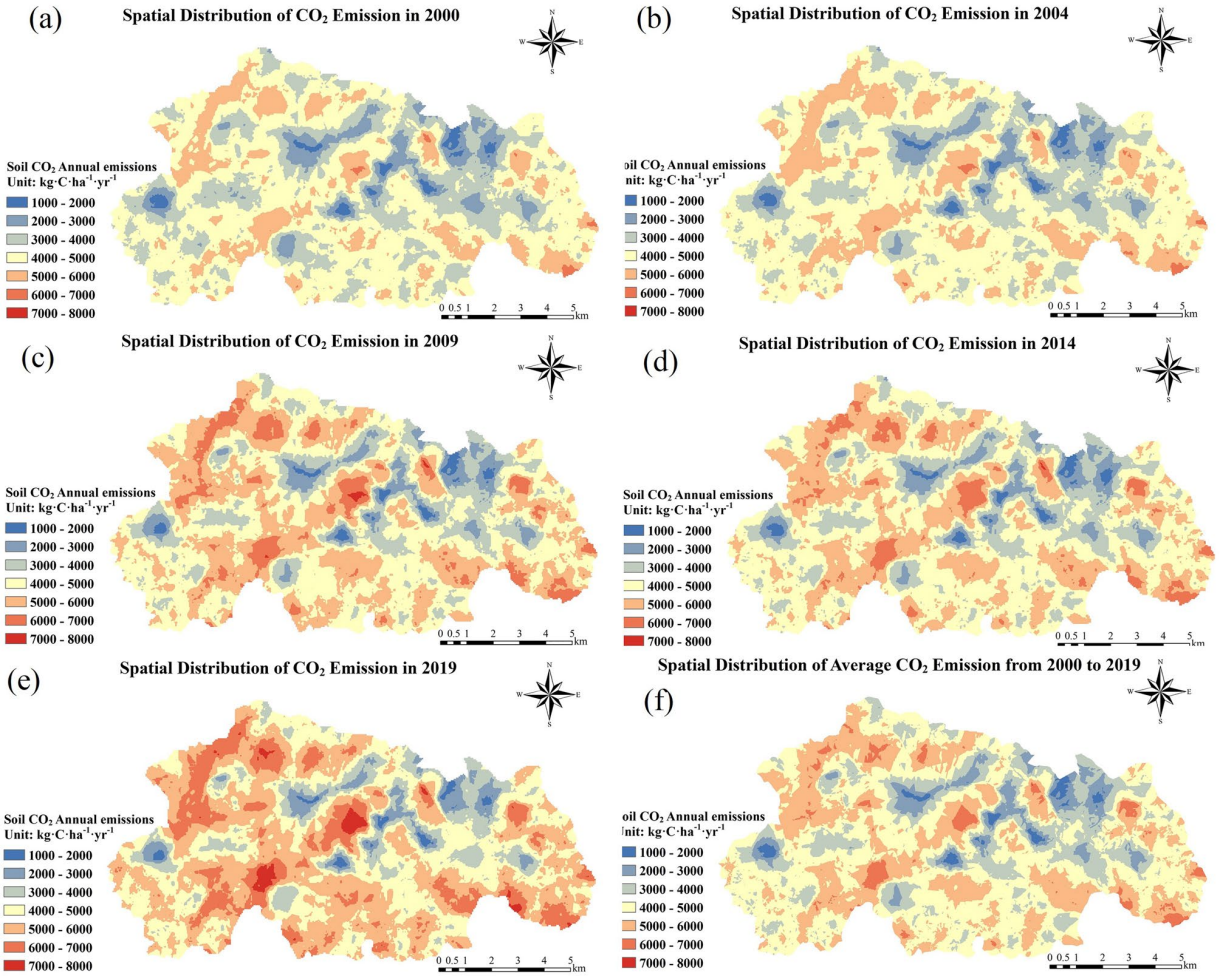
Spatial distribution characteristics of CO_2_ emissions in the Caohai Karst Plateau wetlands over 5-year time intervals and the whole period from 2000–2019.

The spatial pattern of soil CO_2_ emissions in the Caohai wetland (Fig. 4, Supplementary Table S2) showed that the areas with higher CO_2_ emission fluxes in the Caohai wetland were mostly around the lake and in the eastern peripheral area. The lower value area was mostly in the Caohai Lake district and its northeastern region. In general, most areas of the Caohai wetland (approximately 70.96%) were in the median range (4.0 ~ 6.0 t·C·ha^−1^·yr^−1^), while small areas were in the low range (approximately 25.97%, 1.0 ~ 4.0 t·C·ha^−1^·yr^−1^) or high range (approximately 3.07%). 6.0–8.0 t·C·ha^−1^·yr^−1^) (Supplementary Table S2). The major LC types in the low, medium, and high ranges were cropland (60.31%, 65.17%, 54.05%, respectively), water (18.04%, 15.57%, 23.58%, respectively), and forest (13.50%, 12.28%, 14.46%, respectively). The results showed that cropland, water, and forest were the major LC types that affected the CO_2_ emission flux of the Caohai wetland. Therefore, controlling the CO_2_ emissions of these three LC types is the key to controlling the total CO_2_ emissions of the Caohai wetland.

**Figure 5:**
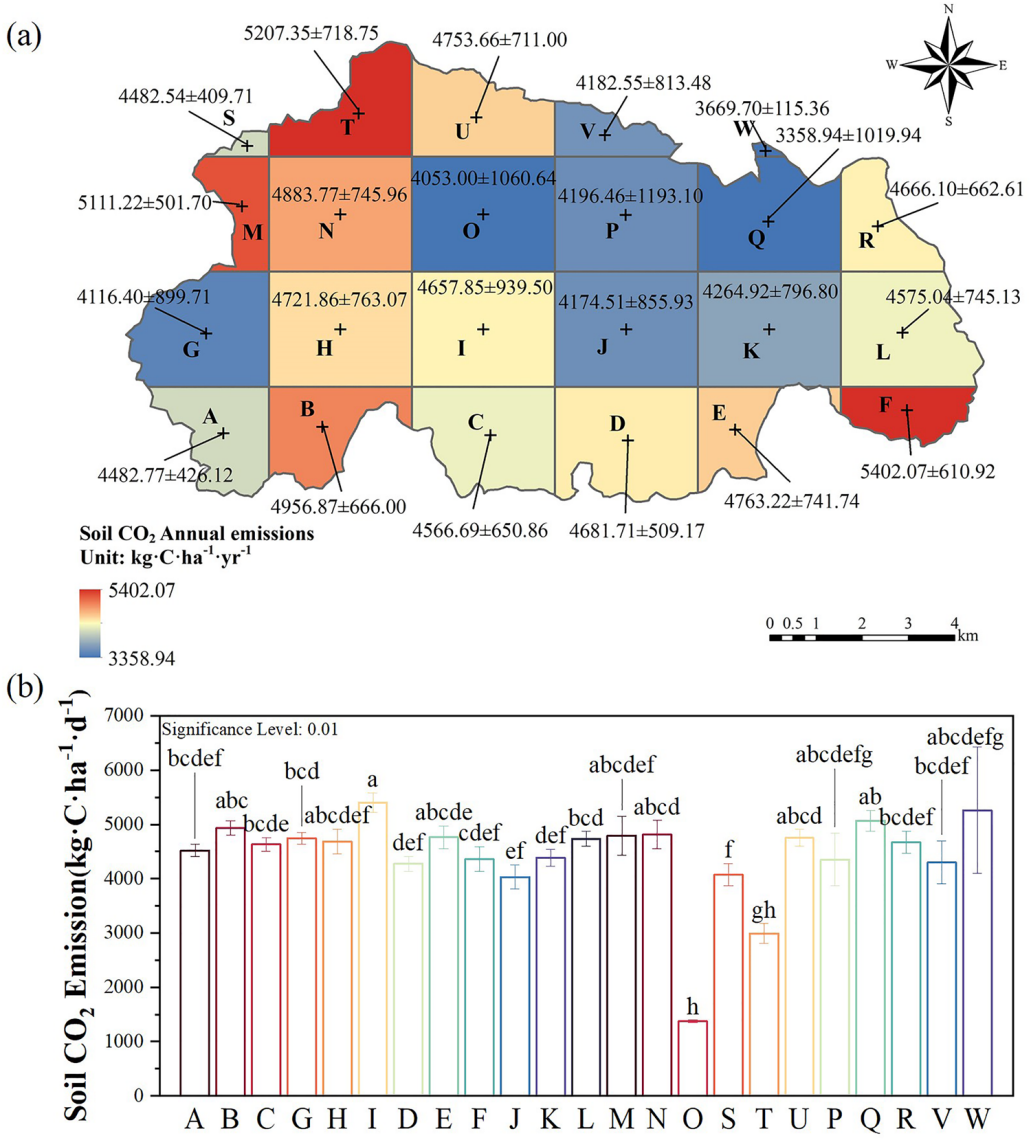
Comparison of spatial variation characteristics of CO_2_ emissions.

**Figure 6:**
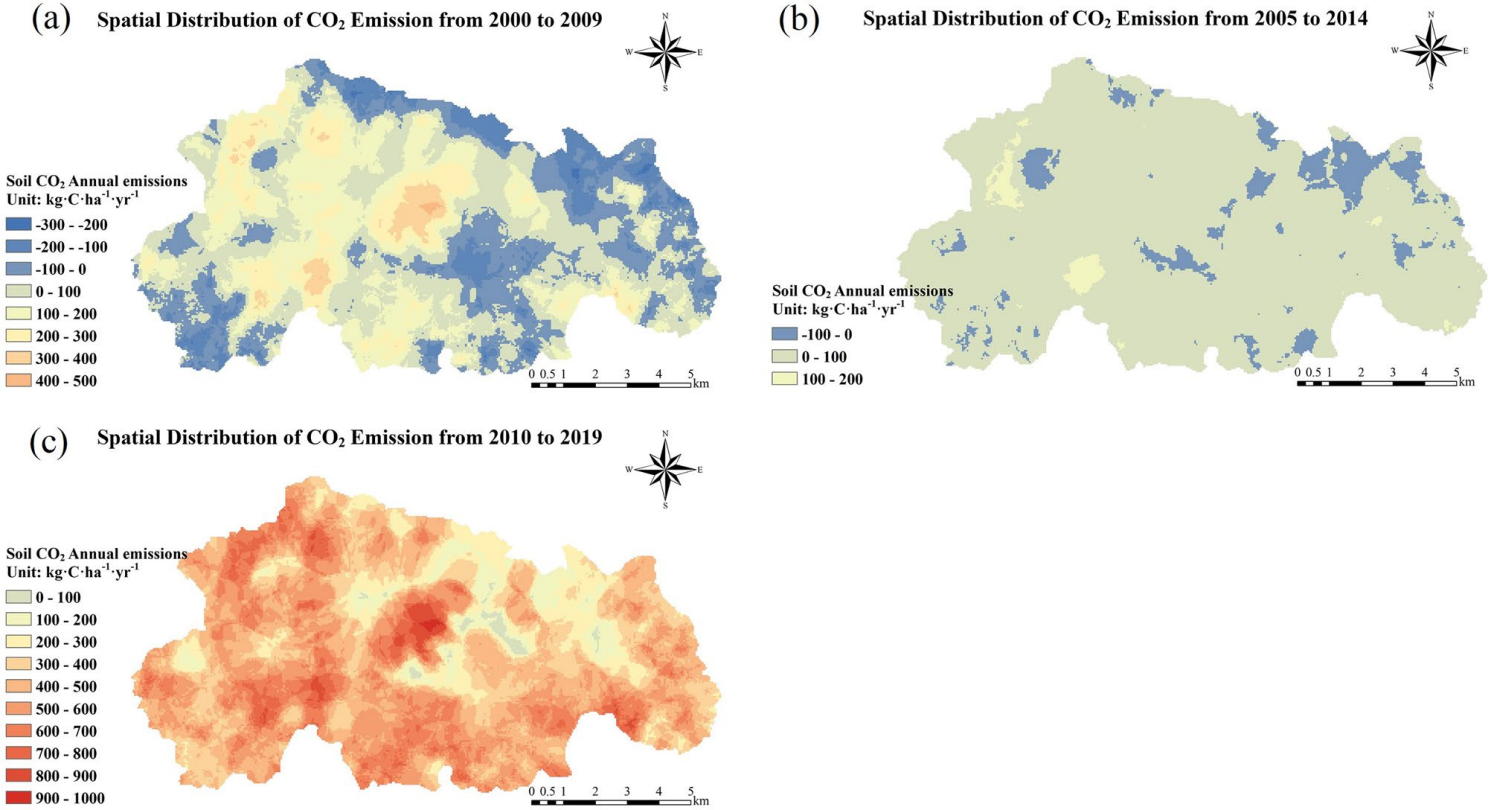
Interannual spatial distribution of the MAC emissions in Caohai. We drew this set of diagrams in the following steps. The grid data of the soil MAC emission flux of 5 years in each group was taken, and then, the grid data of the second group were subtracted from the first group to obtain (**a**), and the average value of the third group was subtracted from the second group to obtain (**b**). The average of the fourth group was subtracted from the third group to obtain (**c**). Positive values in the figure indicate an increase in soil CO_2_ emissions, while negative values indicate a decrease.

The interannual variation of the mean annual CO_2_ (Hereinafter referred to as MAC) emissions in the Caohai wetland generally showed an increasing trend (Figs. 4, 5, 6). We divided the study period (2000–2019) into four groups on average and analyzed the variation in CO_2_ emission flux before and after every 5 years for each group. The soil CO_2_ emissions exhibited an overall decreasing trend over the five years from 2009 to 2014 (the decreasing amount was 0–400 kg·C·ha^−1^·yr^−1^), and the remaining periods (2000–2004, 2005–2009, 2015–2019) exhibited an overall increasing trend. The increases in cropland, water, and forest were 0–300, 0–1100, and 0–000 kg·C·ha^−1^·yr^−1^, respectively.

### Single-factor variation

**Figure 7:**
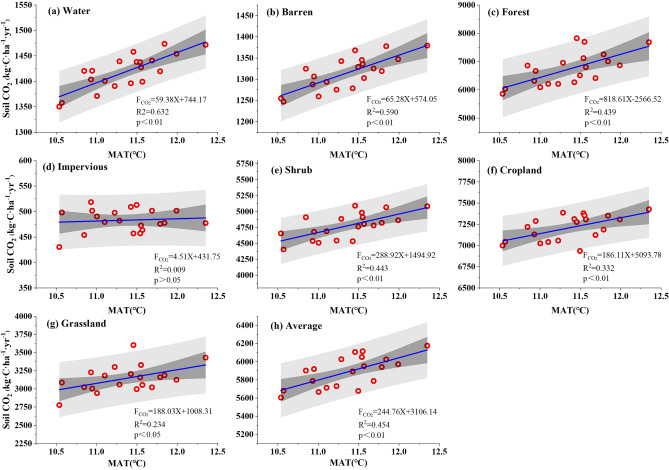
Response of the MAC emissions to MAT in the Caohai wetland as a whole and for each landcover type.

The single-factor regression analysis results (Fig. 7, Supplementary Table S3) showed that there was a significant positive correlation (0.674**) between soil CO_2_ emissions and temperature in the Caohai wetland. Specifically, the MAC emissions increased by 244.756 kg·C·ha^−1^·yr^−1^ with every 1 °C rising in MAT. The average soil CO_2_ emissions of each LC type also increased with increasing temperature. The response degrees of each LC type to temperature were as follows: water (0.795**) > barren land (0.768**) > shrub (0.666**) > forest (0.663**) > cropland (0.576**) > grassland (0.484**) > impervious land (0.092). Except for impervious cover, temperature variation very significantly impacted the CO_2_ emissions of other LC types.

**Figure 8:**
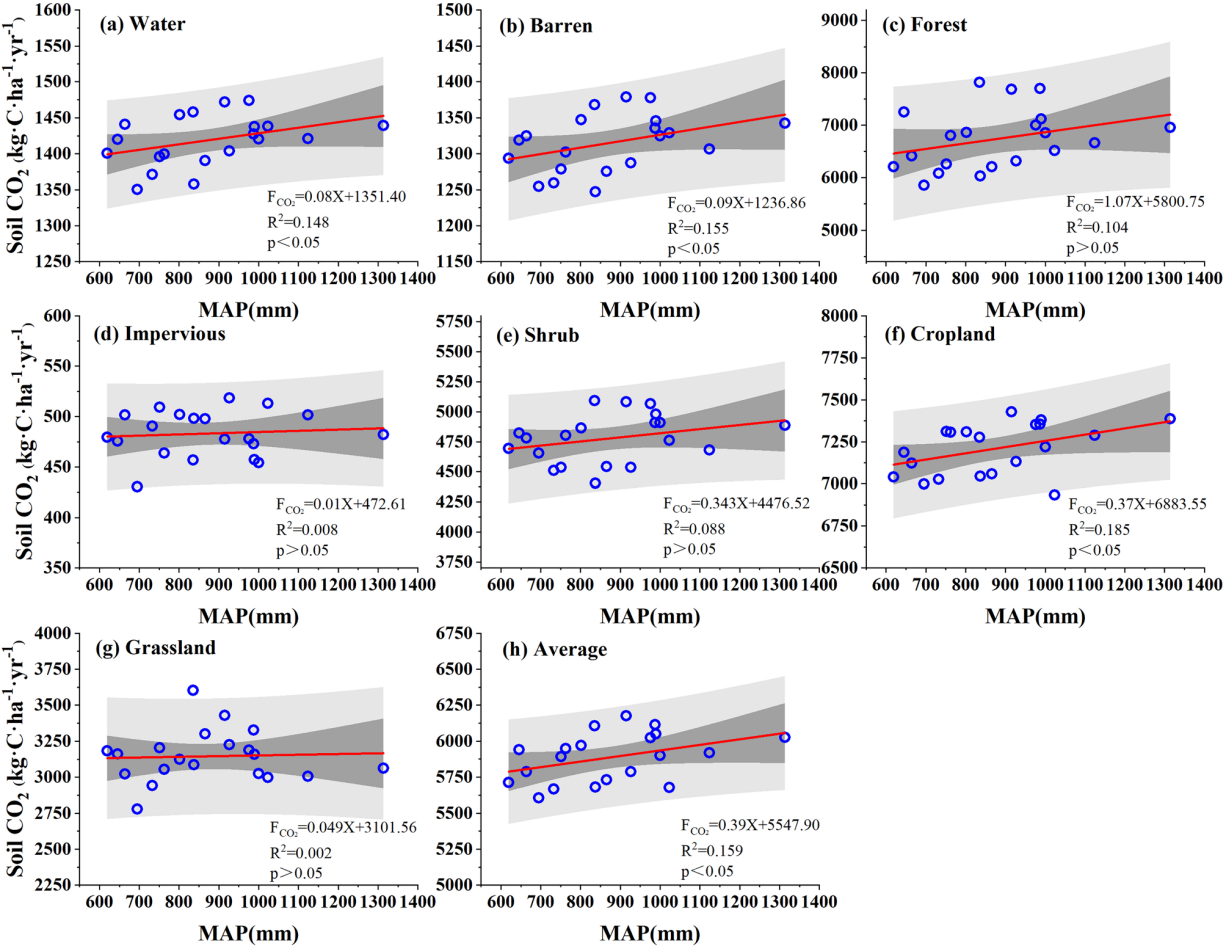
Response of the MAC emissions to MAP in the Caohai wetland as a whole and for each landcover type.

The single-factor regression analysis results (Fig. 8, Supplementary Table S3) showed that there was a positive correlation (0.399*) between soil CO_2_ emissions and precipitation overall. Specifically, the MAC emissions increased by 0.387 kg·C·ha^−1^·yr^−1^ for every 1 mm rising in MAP. All the LC types exhibited patterns similar to those of the Caohai wetland overall, revealing a trend towards increasing soil CO_2_ emissions with increasing precipitation. The variation in the response of each LC type to precipitation decreased in the order of cropland (0.430*) > barren land (0.394*) > water (0.385*) > forest (0.323) > shrub (0.296) > impervious land (0.091) > grassland (0.047). The LC types with the highest and lowest responses to precipitation were barren land and grassland, respectively.

The simulation results revealed that the single-factor model for the temperature of each LC type was strongly correlated, while only a small part of the simulation results of the single-factor model for precipitation were significant. These findings indicated that the soil CO_2_ emissions in the Caohai wetland may be significantly controlled by temperature, while it is not sensitive to the control of precipitation. By comparing and analyzing the single-factor correlation coefficients of soil CO_2_ emissions to temperature and precipitation, we can conclude that the response of soil CO_2_ emissions to climate factors differs and that temperature can explain 45.4% of the variation, while precipitation can explain 15.9%. In general, the soil CO_2_ emissions in the Caohai wetland were controlled by temperature and precipitation and were more easily controlled by temperature.

### Two-factor variation

**Figure 9:**
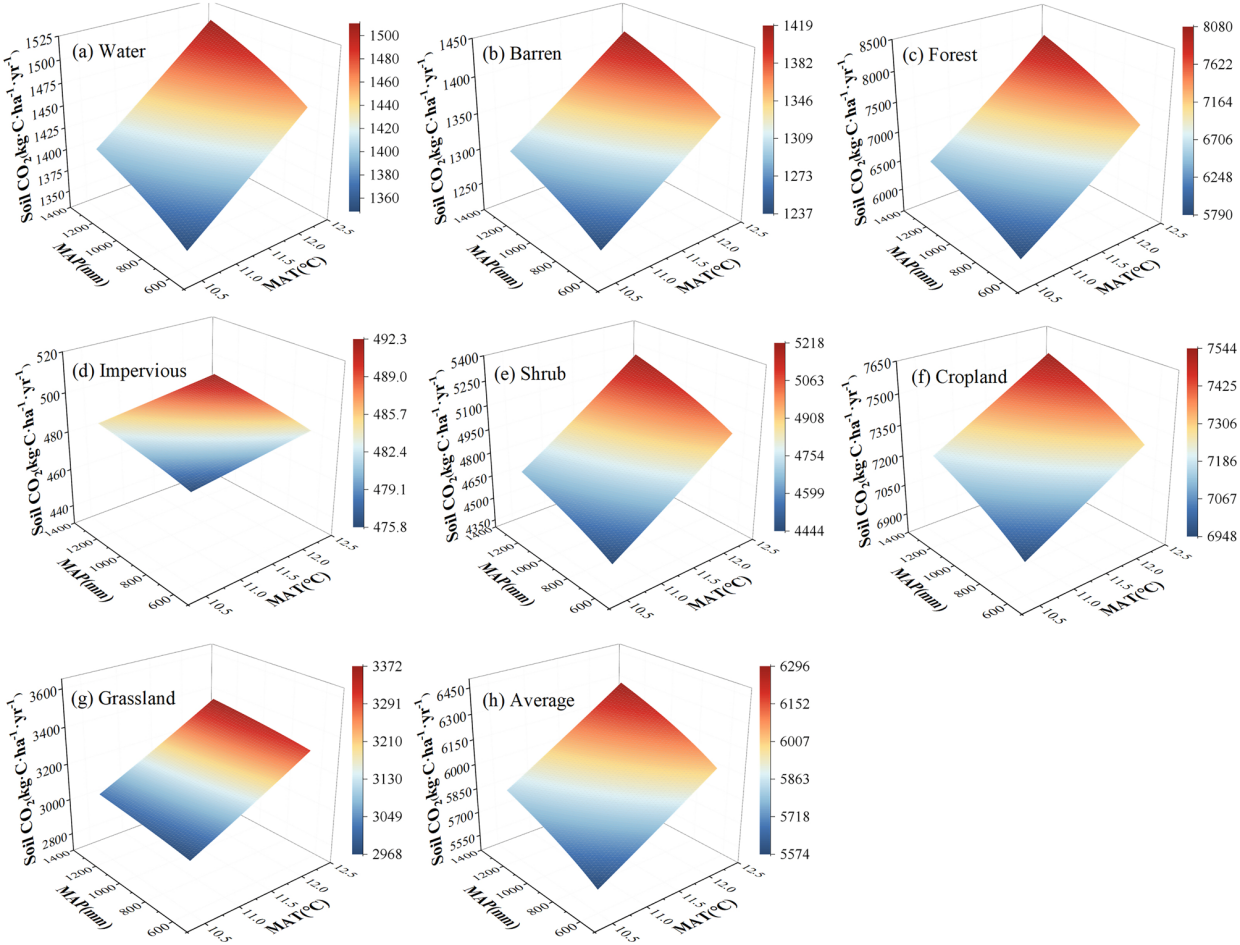
Response of the MAC emissions of the Caohai wetland as a whole and each landcover type to the interaction between temperature and precipitation.

The soil CO_2_ emission flux in the Caohai wetland exhibited a significantly positive correlation with the combined effects of temperature and precipitation, with a correlation coefficient of 0.79 (Fig. 9, Supplementary Table S4). The variation patterns of each LC type were water (0.89**) > barren land (0.87**) > average (0.79**) > forest (0.74**) > shrub (0.73**) > cropland (0.72**) > grassland (0.49**) > impervious land (0.13**) (Supplementary Table S4). The combined effect explained 63% of the variation in soil CO_2_ emissions. The data of the two models showed that the two-factor model can better reflect the feedback of soil CO_2_ emissions to climate change in the Caohai wetland.

### Tillage

**Figure 10:**
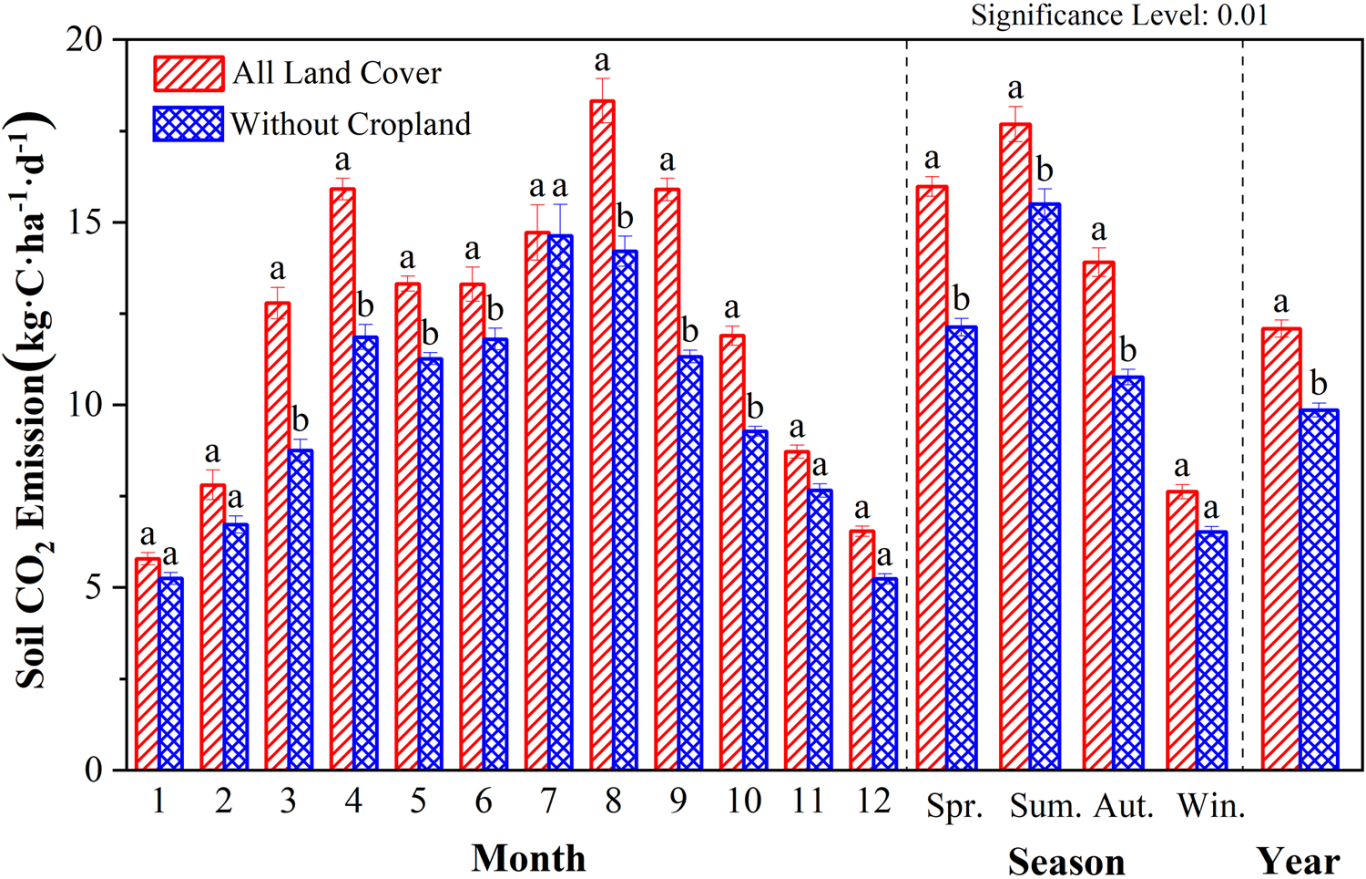
Comparison of the annual variation characteristics of the soil MAC emissions in the Caohai wetland.

Cropland is the LC type affected by field management in various LC types and is relatively affected by anthropogenic factors. Therefore, we compared the soil MAC emissions overall with excluding cropland, and the results are shown in Fig. 10. After removing cropland, the highest emission month changed from August to July, and the soil CO_2_ emission flux also decreased significantly in April. Anthropogenic factors (cropping) significantly impact soil MAC emissions and can lead to a significant increase in Caohai wetland.

### Cropland conversion

**Figure 11:**
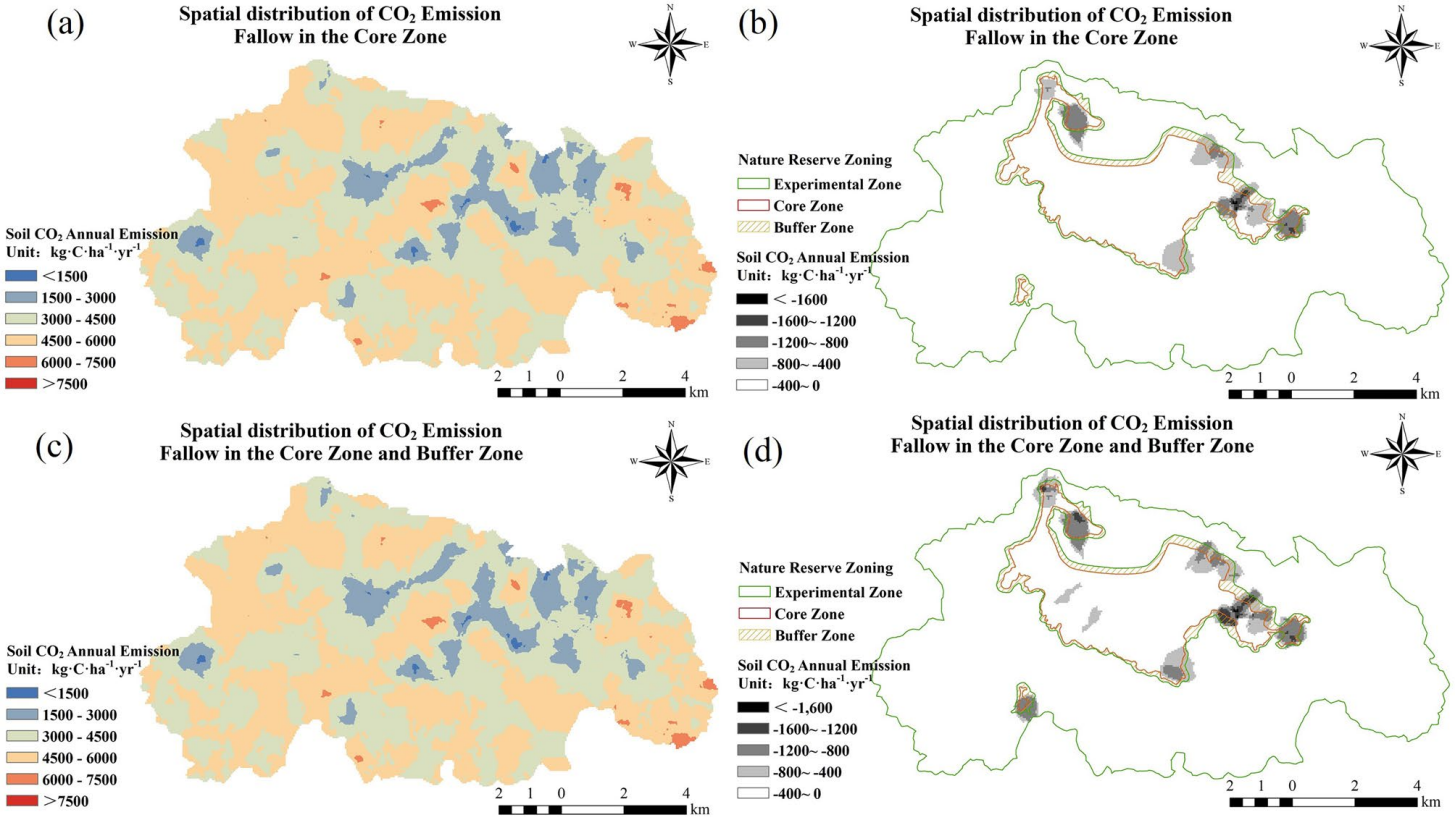
Impacts of cropping on soil MAC emissions in the Caohai wetland under the two scenarios. Figures (**a**) and (**b**) show the changes in soil MAC emissions after no-cropping only in the core zone, and Figure (**c**) and (**d**) show the changes after no-cropping in the core zone and buffer zone.

Agricultural production activities (cultivation) exacerbate soil CO_2_ emission intensity in the Caohai Plateau wetland (Fig. 10). Thus, we have the following question: how can returning farmland to lakes and grasslands affect soil CO_2_ emissions in these regions? To answer this, we simulated and analyzed the situation after different areas of the Caohai wetland were converted to grassland. The results showed that under the scenario in which the cropland in the core zone and the buffer zone were returned to grassland, the soil CO_2_ emission flux of the Caohai wetland decreased significantly in all regions (Fig. 11). The soil carbon emissions of the Caohai wetland could be reduced by 625.78 t·C·yr^−1^ under the scenario in which the cropland in the core zone returned to grassland (Fig. 11a,b, Supplementary Table S5). Under the scenario in which both the core zone and the buffer zone were reduced, the Caohai wetland soil carbon emissions could be reduced by 1002.56 t·C·yr^−1^ (Fig. 11c,d, Supplementary Table S5). This indicated that human factors and land use/cover changes can significantly affect soil CO_2_ emissions in the Caohai Plateau wetland, and a reasonable reduction in cropland area can significantly reduce soil CO_2_ emissions. Therefore, to cope with global warming, reasonable regulation of cropland area in the study areas is necessary. Appropriate human participation positively affects reducing GHGs emissions.

## Discussion

The results indicated that the soil MAC emissions of the Caohai plateau wetland as a whole and of all the other LC types were positive. Thus, the soil pool of the Caohai Plateau wetland was a CO_2_ emission source overall, the CO_2_ emission flux was approximately 5.89 ± 0.17 t·C·ha^−1^·yr^−1^, and the total CO_2_ emission was approximately 70.62 ± 2.04 Gg·C·yr^−1^ (approximately 12 thousand hectares), with high spatial heterogeneity. Due to differences in research methods and climate, soil, and water conditions, the soil CO_2_ emission flux in plateau wetlands is highly variable. Researchers have shown that the CO_2_ emission flux of plateau wetlands is approximately 0.80–8.08 t·C·ha^−1^·yr^−1^ (Table 2). Our results were consistent with existing studies. However, the DNDC model calibrated after long-term continuous observation data was used in this study to simulate the soil CO_2_ emissions of the Caohai Plateau karst wetland; this approach increased the reliability of the results and promoted scale expansion from the point scale to the basin scale, and the results were more general and representative.

**Table 2:** Comparison of soil CO_2_ emission fluxes in plateau wetlands in different regions.

Region	Method	Research period	Type	CO_2_ emission flux kg·C·ha^−1^·yr^−1^	References
Tibetan plateau	CS	2012–2014	Tibetan Plateau fen	1289–1933	^34^
			Tibetan Plateau wet meadow	2815–3429	
			Plateau meadow	6634–7091	
Qinghai-Tibetan plateau	EC	2004	Alpine wetland meadow	6768	^33^
		2005		7264	
		2006		8082	
Bayinbuluk alpine wetland	ACS		Alpine wetland	6780	^35^
Qilian mountains	EC	2014–2016 growing season	Alpine wetland	4059 ± 89	^29^
Qinghai lake	EC	2011.07–2013.06	Alpine wetland	3954.5	^36^
Chinese loess plateau	CS	2019	Semiarid hard-water reservoir	1124.2–6398.45	^37^
Tibet plateau	headspace equilibration method	2019–2021	Thermokarst lakes	1433.46	^38^
Lake Taihu	headspace equilibration method	2000–2015	Lake	797.16	^39^
Caohai wetland	ACS + DNDC	2000–2019	Alpine karst wetland	5886.07 ± 170.27	This study

*EC* means eddy covariance, *CS* means chamber system, and *ACS* means automatic chamber system.

Our results indicated that the alpine wetland represented by the Caohai Plateau Karst wetland has a high CO_2_ emission potential. Its harsh natural environment (high altitude, low temperature, etc.) leads to the easy release of a large amount of carbon due to climate warming (Figs. 7, 9). Most of the world’s wetlands are at high latitudes in the Northern Hemisphere and are mostly covered by permafrost^40,41^. The permafrost stores about 1700 Pg C, which is approximately twice as large as the atmospheric carbon pool^7,42^. Many studies have shown that the carbon stored in permafrost can be easily affected by climate warming and permafrost degradation, leading to its release into the atmosphere^2,43,44^. The greenhouse effect caused by carbon released into the atmosphere will further aggravate global warming; thus, a positive feedback phenomenon occurs^3,45^. At present, reversing the increase in carbon emissions due to the melting of permafrost is difficult, and many scientists are focused on the distribution of permafrost and the change in carbon emission patterns in northern high-latitude regions^46–48^. Our results also prove that ice and snow melt lead to a short-term increase in soil CO_2_ emissions (Table 1). Therefore, controlling the release of soil carbon in alpine wetland ecosystems is essential for reducing GHGs emissions and slowing down the global warming tendency. Correctly regulating the impact of anthropogenic factors on wetland ecosystems through measures and effectively reducing wetland GHGs emissions is an effective way to reduce global GHGs emissions, slow greenhouse effects, and achieve the zero-emissions goal and 2 °C temperature targets of the Paris Agreement.

Temperature and precipitation are important indicators of climate change. Although many biological and abiotic factors affect soil CO_2_ emission intensity, many studies have shown that soil CO_2_ emissions were strongly correlated with temperature and precipitation^13,20,49,50^. In this study, based on DNDC model simulations, soil MAC emissions in the Caohai Plateau wetland showed a highly significant positive correlation with the MAT, and the correlation coefficient was 0.674, which strongly proved that soil CO_2_ emissions in plateau wetlands exhibited a very significant positive correlation with temperature. Our results were consistent with many studies^14,51,52^. At present, scholars have also summarized a variety of equations to quantitatively describe soil respiration intensity change with temperatures^53–57^. Temperature promotes the decomposition of reaction substrates by affecting plant activities and the metabolism and activities of microorganisms in the soil, thus increasing soil CO_2_ emissions^58^. Wetland soil CO_2_ emission fluxes often increase with increasing temperature, and this trend is extremely obvious in the plateau wetlands studied here (Fig. 7). Similarly, a warming experiment conducted by scholars in the alpine steppe ecosystem of northern Tibet showed that the soil CO_2_ flux increased with the temperature rising (temperature sensitivity coefficient was 1.41)^22^. In the Appi Highlands secondary forest, soil CO_2_ emissions increased exponentially with temperature, with a temperature sensitivity of 3.7^59^. In this study, with each temperature increase of 1 °C, the soil CO_2_ emission flux increased by 244.756 kg·C·ha^−1^·yr^−1^, R^2^ was 0.454, and the correlation coefficient was 0.674 (Fig. 7), indicating that temperature variation explained 45.4% of the soil CO_2_ emission change in the Caohai Plateau wetland. Therefore, temperature may be the dominant factor influencing soil CO_2_ emissions in the Caohai wetland. At present, global warming is intensifying. Warming will exacerbate CO_2_ emissions, and an increase in CO_2_ emissions as a GHGs will further aggravate global warming, forming a positive feedback phenomenon. Therefore, controlling and reducing GHGs emissions exhibits an extremely essential positive effect on global climate change and can reduce the extreme weather and subsequent disasters caused by global warming, which will be the goal of future work.

Second, precipitation was another controlling factor in our research. According to the simulation results, the soil MAC emissions in the Caohai Plateau wetland exhibited a positive correlation with the MAP, for which the correlation coefficient was 0.399. Previous studies have confirmed the rationality of these results^23,51,60^. Research has shown that precipitation affects gas diffusions in soil and limits the transfer of substances by affecting soil moisture. Moreover, a high soil water content can cause soil hypoxia, resulting in changes in the soil’s physical environment (such as pH and Eh), thereby affecting soil microbial activity, plant root activity, and CO_2_ production and emission^22,61–63^. A large number of experimental data showed that the increase of precipitation usually leads to the increase of soil CO_2_ emissions^48,57,61^. Our results that the soil CO_2_ emissions increased by 0.387 kg·C·ha^−1^·yr^−1^ (R^2^ = 0.16) with a 1 mm rise in precipitation also showed that an increase in precipitation leads to an increase in soil CO_2_ emissions. The precipitation explained 16% of the soil MAC emission variation in the Caohai Plateau wetland, while temperature explained 45.4%. Therefore, compared with temperature, precipitation is the secondary control factor. Furthermore, according to the two-factor model results, the interaction effect (R^2^ = 0.63, R = 0.79) was higher than that of temperature or precipitation. This indicated that it was the interaction that mainly influences the soil CO_2_ emissions variation, while other research results support this view^60,64,65^. In addition, the combined effect is not only a simple superposition effect. Rising air temperature usually results in soil temperature rising, but it results in soil moisture decreasing too. Increasing precipitation leads to soil moisture increasing and soil temperature decreasing^66^. Therefore, when we are coping with global climate change, temperature, and its interaction should be the main climate-influencing factors that receive attention. In addition, the interaction between different climate factors is another way to slow global warming trends. Additional joint influences of climate factors should be included in future research on climate.

Anthropogenic factors are one of the important reasons for the variation in CO_2_ emissions in wetlands. The simulation data based on the DNDC model in this study showed that anthropogenic factors (cropping and land use/cover change (LUCC)) increased the flux and total emissions from wetland soil, and these results are consistent with existing research results^67–69^. Anthropogenic factors (such as LUCC and the input of exogenous nutrients) can affect the quality and amount of reaction substrates required for life activities, change the soil condition, and cause changes in the microorganisms’ abundance and life activity intensity, leading to variations in CO_2_ emissions^4,21,70^. Studies have shown that anthropogenic nitrogen input increased soil respiration by 14% in two years on the Loess Plateau^25^. The effects of multiple levels of nitrogen input on wetland ecosystem respiration in the Northeast Sanjiang Plain were 28–69%^71^. In the past 300 years, the expansion of cultivated land in Northeast China has generated 1.06–2.55 Pg C emissions^72^. Similarly, in this study, cultivation caused additional peak soil CO_2_ emissions in the Caohai wetland and the transfer of the month with the highest emissions (Fig. 10). In the future, the return of cropland in the core zone could reduce soil CO_2_ emissions by 625.78 t·C per year. Moreover, under the scenario in which both the core zone and the buffer zone are fallowed, the soil CO_2_ emissions of the wetland can be reduced by 1002.56 t·C·yr^–1^ (Fig. 11). However, studies have shown that regulated deficit irrigation (RDI) measures can reduce the average soil CO_2_ emissions by 1088–1664 g·CO_2_·m^−2^^73^. The application of soil amendments such as biochar can significantly reduce soil CO_2_ emissions^74^. Therefore, anthropogenic factors can either enhance or reduce CO_2_ emissions from wetlands. In addition, from the existing studies, we can find that reasonable planning of wetland areas, "returning ponds/plowing to lakes and grasslands", reasonable human intervention, and control of chemical substances produced by human production and life in wetlands can effectively reduce CO_2_ emissions in wetlands. In summary, anthropogenic factors can greatly change the CO_2_ emissions of wetlands, and the CO_2_ emission process of wetland ecosystems is highly susceptible to the influence and regulation of anthropogenic factors. Therefore, reasonable anthropogenic factors can effectively control the CO_2_ emissions of wetlands while maintaining the ecological function value of wetland ecosystems, thus reducing the warming potential of wetland CO_2_ emissions, which can reduce carbon emissions and mitigate climate change.

## Methods

### Study area

**Figure 12:**
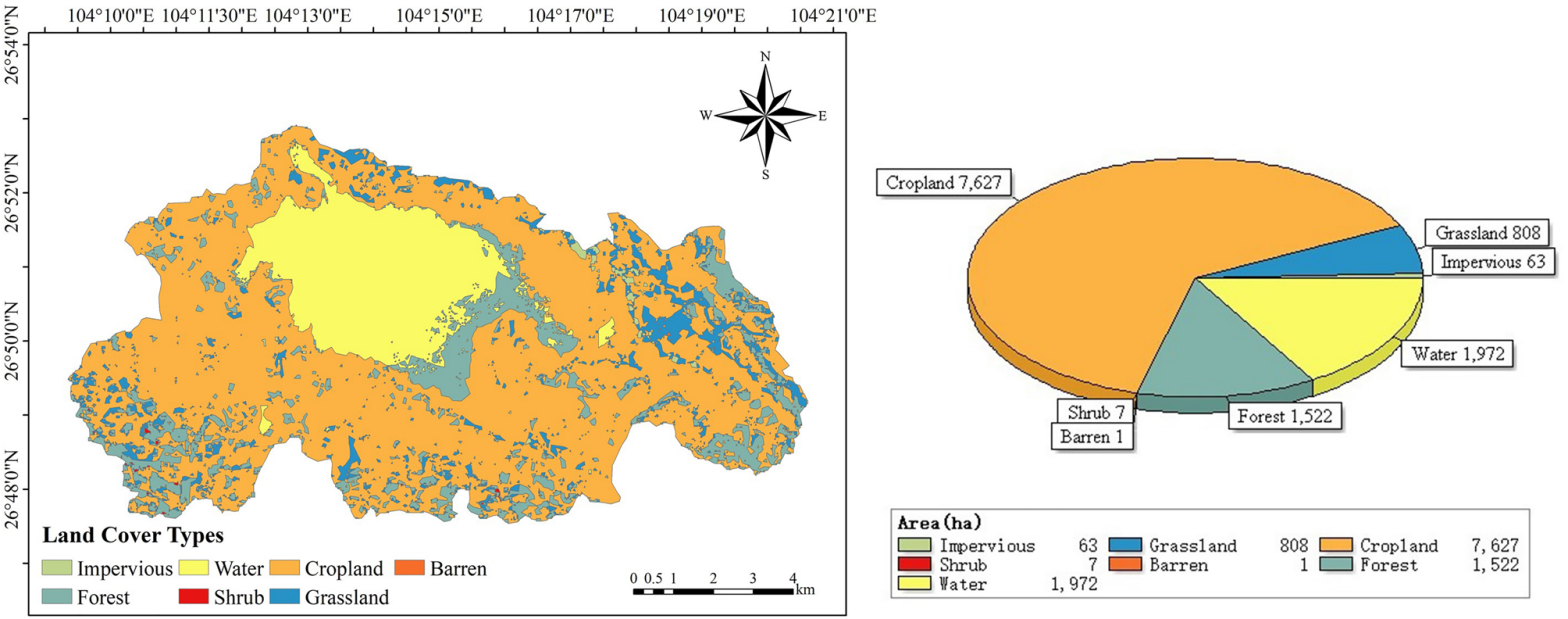
Spatial distribution and area of land cover types in Caohai.

The research site was the Caohai National Nature Reserve of Guizhou Province (hereinafter referred to as Caohai wetland), which lies south of Weining County, Guizhou Province (26° 47′ 35″ N–26° 52′ 10″ N, 104° 9′ 23″ E–104° 20′ 10″ E, 2200–2250 m) (Fig. 12). The Caohai wetland is the source lake of the Luoze River, a tributary of the Yangtze River. It is a typical karst lake formed under the influence of geological structure, with a normal water storage area of 1980 ha (corresponding water level is 2171.7m). Caohai Lake's water depth varies greatly, the deepest can reach approximately 5 m^75^. The Caohai wetland is located in a subtropical zone and has a typical monsoon climate with distinct characteristics of dry winters and wet summers. The mean annual temperature (MAT) in this region is 10.9 °C, the hottest month (July) is 17.3 °C, and the coldest month (January) is 2.1 °C. The mean annual precipitation (MAP) is 903.6 mm, which occurs mainly from May to August (accounts for 70.4% of the total). The monthly mean maximum evaporation is 45.8–117.6 mm, and the annual mean evaporation is 948.7 mm. Evaporation is mainly concentrated from March to May. The annual sunshine duration ranges from 1374 to 1633.7 h, and the annual mean sunshine duration is 1455.5 h^76^.

### Data source and preprocessing

Meteorological, land cover, soil, and other data were mainly applied in this study (Table 3). After coordinating projection transformation (WGS_1984_UTM_Zone_48N), mask extraction, partition statistics, and other operations, all raster data were imported into patches of appropriate size and converted into .txt files for subsequent model operations.

**Table 3:** Data information and sources.

Data	Time	Resolution	Source
Meteorological data	2000–2020	1 d	China Meteorological Data Service Centre (http://data.cma.cn/)
Land cover data	2000–2020	30 m	(10.5194/essd-13-3907-2021)
Nitrogen deposition data	1980–2100		(10.12199/nesdc.ecodb.mod.2021.010)
Soil data	−	250 m	Loess Plateau Science Data Centre, National Earth System, Science Data Sharing Infrastructure, National Science & Technology Infrastructure of China (http://loess.geodata.cn)

### Model estimation

In this study, four statistical parameters (the relative mean absolute error (RMAE), relative root mean square error of prediction (RRMSE), coefficient of determination (R^2^), and Nash–Sutcliffe model efficiency coefficient (E); Eqs. (1–4)) were used to calibrate the simulation accuracy and reflect the simulation effect in this study area^77–79^.

1 $$RMAE=\frac{{\sum }_{i=1}^{n}\left|{x}_{i}-{y}_{i}\right|}{n}/\overline{x },$$

2 $$RRMSE=\sqrt{\frac{{\sum }_{i=1}^{n}{\left({y}_{i}-{x}_{i}\right)}^{2}}{n}}/\overline{x },$$

3 $$R=\frac{{\sum }_{i=1}^{n}\left({x}_{i}-\overline{x }\right)\left({y}_{i}-\overline{y }\right)}{\sqrt{{\sum }_{i=1}^{n}{\left({x}_{i}-\overline{x }\right)}^{2}{\sum }_{i=1}^{n}{\left({y}_{i}-\overline{y }\right)}^{2}}},$$

4 $$E=1-{\sum }_{i=1}^{n}{\left({x}_{i}-{y}_{i}\right)}^{2}/{\sum }_{i=1}^{n}{\left({x}_{i}-\overline{x }\right)}^{2},$$

where n is the number of samples, ${x}_{i}$ is the measured data, and ${y}_{i}$ is the model simulation result data.

### Regression model analysis

A regression model is a mathematical modeling technique that quantitatively describes the relationship between different variables. This technique is widely used in forecasting and modeling. In this study, both the single-factor linear regression model (Eq. (5)) and the two-factor nonlinear regression model (Eq. (6)) were used to compare and analyze the response of soil CO_2_ emissions to temperature and precipitation, respectively^80,81^.

5 $${F}_{{CO}_{2}}={\text{aX}}+{\text{b}},$$

6 $${F}_{{CO}_{2}}={\text{a}}\cdot {\text{exp}}\left(b\cdot T\right)\cdot {P}^{c}$$

where a, b, and c are fitting parameters, ${F}_{{CO}_{2}}$ represents the annual mean CO_2_ emission flux (kg·C·ha^−1^∙yr^−1^), the units of T and P are ℃ and mm, respectively, and X represents the MAT or MAP.

### Model accuracy verification results

**Figure 13:**
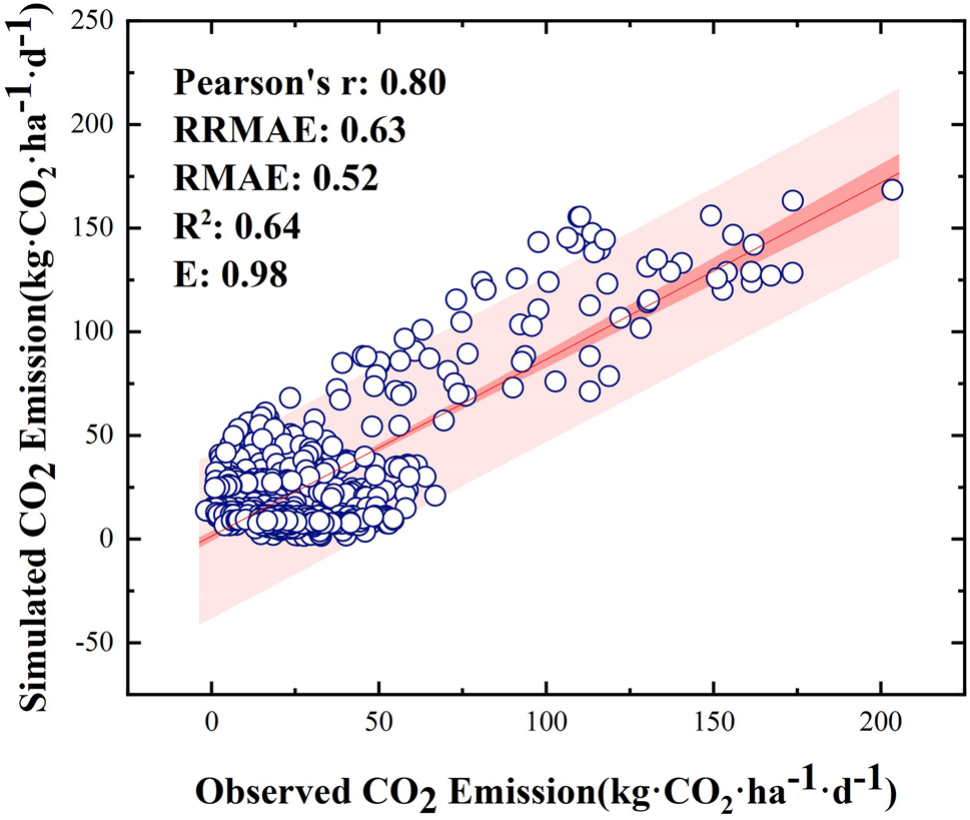
Accuracy verification of the observed and simulated data.

In this study, the observed daily soil CO_2_ flux data measured continuously by an LI-8150 multichannel auto-chamber system were used to drive and verify the reliability of the DNDC model. Between the simulated data and the observed data, the Pearson correlation coefficient (P) was 0.81, the RRMSE was 0.61, the RMAE was 0.50, the R^2^ was 0.69, and the E value was 0.98 (Fig. 13). The data showed that it is of high feasibility and reliability to use the DNDC model to simulate soil CO_2_ emissions in plateau karst wetlands. This model can accurately reflect the soil CO_2_ emission situation in the Caohai wetland.

## Conclusion and prospects

In the context of global change, the study aimed at major GHGs in wetland soil is highly important for assessing regional GHGs budgets and their response to environmental change. Based on the DNDC model, the spatiotemporal variations in soil CO_2_ emissions in the Caohai wetland from 2000 to 2019 were analyzed in combination with long-term daily soil CO_2_ respiration data, meteorological data, and remote sensing data. The effects of meteorological factors (temperature and precipitation) and anthropogenic factors on soil CO_2_ emissions were discussed. Our preliminary conclusions are as follows.

- The soil CO_2_ emissions in the Caohai Karst Plateau wetland are large, with an average value of 5.89 ± 0.17 t·C·ha^−1^·yr^−1^, and the average total soil CO_2_ emissions are 70.62 Gg·C·yr^−1^ (study area: approximately 12 thousand hectares), with high spatial heterogeneity ranging from 1.13 to 7.09 t·C·ha^−1^·yr^−1^. The CO_2_ emission flux of cropland was the highest, followed by that of forest, and the lowest was that of impervious land.
- In terms of temporal changes, soil CO_2_ emissions in the study area increased annually, with an annual growth rate of 23.02 kg·C·ha^−1^·yr^−1^ in the past 20 years (2000–2019). Spatially, the soil CO_2_ emissions around the Caohai Lake area were the highest, while the radiation to the surrounding area decreased.
- Soil MAC emissions are sensitive to climate change. Temperature and precipitation change jointly account for approximately 63% of the variation in soil CO_2_ emissions. However, temperature changes are more important than precipitation changes for soil CO_2_ emissions in the Caohai Plateau wetland, which accounts for approximately 45.4%. Anthropogenic factors (cultivation and fertilization) significantly increased the soil MAC emission intensity.

In this study, we obtained a series of analysis results on the spatio-temporal patterns in CO_2_ flux in the Caohai Plateau wetland and its response to climate change and revealed the change rules under current and future conditions and with the influence of climate change and anthropogenic factors. The expansion of soil CO_2_ emissions to the basin was assessed on a two-dimensional scale. These results support international and domestic policies and provide data support and method references for scientific and reasonable emission reduction and slowing the global warming trend. First, for GHGs, only the CO_2_ with the highest content in the atmosphere was selected for research in this study, while CH_4_ and N_2_O are also important GHGs in the air. If these three major GHGs can be comprehensively studied and analyzed in future studies, the carbon source/sink of the ecosystem can be calculated more comprehensively and accurately. Second, we explored only the CO_2_ emissions of the soil carbon pool in plateau wetland ecosystems. By combining vertical scale research methods (such as vorticity correlation systems and UAV-based hyperspectral radar/Lidar), a more comprehensive and systematic study of the carbon budget and major GHGs (CO_2_, CH_4_, and N_2_O) of the entire ecosystem can be achieved. Thus, the carbon budget of plateau karst wetland ecosystems can be calculated more reliably and accurately.

### Supplementary Information

Supplementary Tables.

## Data Availability

All data generated or analysed during this study are available upon reasonable request from the corresponding author or Hongyu Jia.
