# Supplementary material for: Soil respiration and its response to climate change and anthropogenic factors in a karst plateau wetland, southwest China
Source: Sci Rep. 2024 Apr 15;14:8653. doi: 10.1038/s41598-024-59495-5 (PMC11018823; doi:10.1038/s41598-024-59495-5)
Supplement: Supplementary file 1 — Supplementary Tables. [file 41598_2024_59495_MOESM1_ESM.pdf]

Supplementary information

Fluxes and Responses of Soil Respiration to Climate Change in a Karst Plateau Wetland

Supplementary Table S1 Parameters of regression model for interannual variation of CO_2 emissions of different land cover types.
Supplementary Table S2 Spatial proportion of annual CO2 emission fluxes of different land cover types.
Supplementary Table S3 Single factor linear model parameter results of annual CO_2 emission flux and temperature and precipitation in Caohai wetland as a whole and each land cover type.
Supplementary Table S4 Two-factor nonlinear model parameter results of annual CO2 emission flux and temperature and precipitation in Caohai wetland as a whole and each land cover type.
Supplementary Table S5 The reduction of cropland area and total CO_2 emission under the two scenario of conversion.

Supplementary Table S1 Parameters of regression model for interannual variation of CO_2 emissions of different land cover types.

Land cover type	Statistics		Correlation	significance	Regression model parameter					
	Mean value	sd	Pearson correlation	F	B	sd	Intercept	sd	Beta	t
	CO_2 emission flux (unit: kg·C·ha^{-1}·yr^{-1})									
Cropland	7207.65	151.46	0.70**	17.04**	17.85	4.33	−28668.17	8691.32	0.70	4.13**
Forest	6731.23	579.07	0.834**	41.08**	81.62	12.73	−157281.63	25589.69	0.83	6.41**
Shrub	4776.47	203.55	0.92**	95.20**	31.55	3.23	−58629.45	6498.40	0.92	9.76**
Grassland	3143.94	182.09	0.33	2.20	10.17	6.85	−17285.02	13759.70	0.33	1.49
Water	1418.60	35.03	0.64**	12.60**	3.80	1.07	−6215.62	2150.76	0.64	3.55**
Barren	1314.90	39.81	0.78**	28.70**	5.28	0.99	−9285.59	1978.67	0.78	5.36**
Impervious	483.00	22.88	−0.65**	13.08**	−2.51	0.69	5524.27	1394.17	−0.65	−3.62**
Average	5886.07	170.27	0.80**	31.93**	23.02	4.07	−40364.18	8185.06	0.80	5.65**
	Total CO_2 emission (unit: Gg·C·yr^{-1})									
Cropland	54.97	1.16	0.70**	17.04**	0.14	0.03	−218.64	66.29	0.70	4.13**
Forest	10.24	0.88	0.834**	41.08**	0.12	0.02	−239.31	38.94	0.83	6.41**
Shrub	0.03	1.47×10^{-3}	0.917**	95.20**	2.28×10^{-4}	2.30×10^{-5}	−0.43	0.05	0.92	9.76**
Grassland	2.54	0.15	0.33	2.20	0.01	0.01	−13.97	11.12	0.33	1.49
Water	2.80	0.07	0.64**	12.60**	0.01	0.002	−12.26	4.24	0.64	3.55**
Barren	7.00×10^{-4}	2.00×10^{-5}	0.78**	28.70**	2.99×10^{-6}	5.58×10^{-7}	−0.01	1.00×10^{-3}	0.78	5.36**
Impervious	0.03	1.45×10^{-3}	−0.65**	13.08**	1.59×10^{-4}	4.40×10^{-5}	0.35	0.09	−0.65	−3.62**
Total	70.62	2.04	0.80**	31.93**	0.28	0.05	−484.25	98.20	0.80	5.65**

Note: B, non-standardized coefficient; SD, standard error; Beta, standardization coefficient; 1 Gg=10^3 t=10^6 kg=10^9 g.

Supplementary Table S2 Spatial proportion of annual CO2 emission fluxes of different land cover types.

Level	Type	Area(ha)	Space proportion （%）	Level	Type	Area(ha)	Space proportion （%）
1	Impervious	10.82	0.09	2	Impervious	1.23	0.01
	Grassland	3.92	0.03		Grassland	10.43	0.09
	Cropland	65.45	0.55		Cropland	50.35	0.42
	Forest	48.08	0.40		Forest	2.07	0.02
	Water	27.56	0.23		Water	8.96	0.07
	Total	155.83	1.30		Total	73.05	0.61
3	Impervious	38.17	0.32	4	Impervious	10.15	0.08
	Grassland	188.03	1.57		Grassland	372.11	3.11
	Cropland	1761.33	14.70		Cropland	3374.80	28.16
	Shrub	0.87	0.01		Shrub	4.80	0.04
	Forest	370.15	3.09		Barren	0.09	0.00
	Water	525.12	4.38		Forest	659.31	5.50
	Total	2883.67	24.06		Water	791.41	6.60
5	Impervious	2.81	0.02		Total	5212.67	43.50
	Grassland	201.58	1.68	6	Impervious	0.02	1.88×10^{-4}
	Cropland	2166.47	18.08		Grassland	29.05	0.24
	Shrub	1.57	0.01		Cropland	198.44	1.66
	Barren	0.48	3.98×10^{-3}		Shrub	0.00	6.00×10^{-6}
	Forest	384.90	3.21		Forest	53.01	0.44
	Water	532.40	4.44		Water	86.69	0.72
	Total	3290.20	27.46		Total	367.22	3.06
7	Cropland	0.30	2.53×10^{-3}	Total		11983.11	100.00
	Forest	0.17	1.40×10^{-3}				
	Total	0.47	$3.93\times^{-3}$				

Note: "Level n" indicates that CO_2 emissions are between n×1000～(n+1)×1000 kg·C·ha^{-1}·yr^{-1}.

Supplementary Table S3 Single factor linear model parameter results of annual CO_2 emission flux and temperature and precipitation in Caohai wetland as a whole and each land cover type.

Parameter		Correlation	R^2	F	Intercept	sd	Slope	sd	Beta	t
MAT (℃)	Water	0.795**	0.632	30.918**	744.166	121.391	59.380	10.679	0.795	5.56**
	Barren	0.768**	0.590	25.944**	574.047	145.567	65.227	12.806	0.768	5.094**
	Forest	0.663**	0.439	14.113**	−2566.519	2476.946	818.608	217.903	0.663	3.757**
	Impervious	0.092	0.009	0.155	431.749	130.172	4.512	11.452	0.092	0.394
	Shrub	0.666**	0.443	14.318**	1494.920	867.926	288.919	76.354	0.666	3.784**
	Cropland	0.576**	0.332	8.948**	5093.780	707.244	186.113	62.218	0.576	2.991**
	Grassland	0.484*	0.234	5.515*	1008.308	910.213	188.029	80.074	0.484	2.348*
	Average	0.674**	0.454	14.991**	3106.136	718.580	244.756	63.215	0.674	3.872**
MAP (mm)	Water	0.385*	0.148	3.138	1351.403	38.657	0.077	0.043	0.385	1.771
	Barren	0.394*	0.155	3.301	1236.861	43.767	0.089	0.049	0.394	1.817
	Forest	0.323	0.104	2.092	5800.750	655.505	1.066	0.737	0.323	1.446
	Impervious	0.091	0.008	0.151	472.614	27.252	0.012	0.031	0.091	0.388
	Shrub	0.296	0.088	1.727	4476.519	232.543	0.343	0.261	0.296	1.314
	Cropland	0.43*	0.185	4.076	6883.553	163.568	0.371	0.184	0.430	2.019
	Grassland	0.047	0.002	0.039	3101.555	217.533	0.049	0.244	0.047	0.199
	Average	0.399*	0.159	3.405	5547.897	186.742	0.387	0.210	0.399	1.845

Supplementary Table S4 Two-factor nonlinear model parameter results of annual CO2 emission flux and temperature and precipitation in Caohai wetland as a whole and each land cover type.

Parameter		Value	sd	t	R	R^2	F
Water	a	630.71	73.83	8.54**	0.89	0.79	45698.20**
	b	0.04	0.01	7.10**			
	c	0.05	0.01	3.53**			
Barren	a	490.02	75.50	6.49**	0.87	0.75	26429.00**
	b	0.05	0.01	6.42**			
	c	0.06	0.02	3.38**			
Forest	a	619.52	367.90	1.68	0.74	0.55	1808.12**
	b	0.12	0.03	4.11**			
	c	0.15	0.07	2.10			
Impervious	a	371.87	178.32	2.09	0.13	0.02	2706.42**
	b	0.01	0.02	0.38			
	c	0.02	0.06	0.40			
Shrub	a	1525.29	453.69	3.36**	0.73	0.54	7105.81**
	b	0.06	0.01	4.08**			
	c	0.07	0.04	1.87			
Cropland	a	3915.52	580.80	6.74**	0.72	0.52	28379.44**
	b	0.03	0.01	3.47**			
	c	0.05	0.02	2.62*			
Grassland	a	1342.87	695.30	1.93	0.49	0.24	2342.42**
	b	0.06	0.03	2.29*			
	c	0.03	0.06	0.42			
Average	a	2429.03	440.40	5.52**	0.79	0.63	19055.69**
	b	0.04	0.01	4.57**			
	c	0.06	0.02	2.80			

Supplementary Table S5 The reduction of cropland area and total CO_2 emission under the two scenario of conversion.

Level (kg·C·ha^{-1}·yr^{-1})	Reduction of Cropland area （ha）	Space proportion（%）	Reduction of total soil CO_2 emission (t·C·yr^{-1})
	Core zone conversion only		
＜–1600	1.47	0.01	–625.78
–1600～–1200	15.64	0.13	
–1200～–800	181.45	1.51	
–800～–400	321.12	2.67	
0	11487.71	95.67	
Total	～12000	100	
	Core zone and buffer zone conversion		
＜–1600	1.47	0.01	–1002.56
–1600～–1200	48.69	0.41	
–1200～–800	274.48	2.29	
–800～–400	377.06	3.14	
0	11307.93	94.16	
Total	～12000	100	
